# Role of plasma fibrinogen and D-dimer as prognostic biomarkers in patients with non-muscle invasive bladder cancer

**DOI:** 10.1186/s12301-023-00350-w

**Published:** 2023-03-30

**Authors:** Sahil Singla, Apul Goel, Sanjay Mishra, Ravi Lohani, Satya Narayan Sankhwar, Sashi Raj Singh

**Affiliations:** 1 Department of Urology, King George’s Medical University, Lucknow, India; 2 Department of Pathology, King George’s Medical University, Lucknow, India

**Keywords:** Plasma fibrinogen, D-dimer, Prognostic marker, Bladder cancer

## Abstract

**Background:**

We aimed to evaluate the role of plasma fibrinogen and D-dimer as prognostic biomarkers in patients with non-muscle invasive bladder cancer (NMIBC).

**Methods:**

The prospective study included 35 patients (30 males) with newly diagnosed NMIBC with no history of thromboembolic event or anti-coagulant intake or active infection and underwent complete trans-urethral resection between September 2020 and December 2021. Patients with deranged hepato-renal functions, refractory hypertension or diagnosed with COVID-19 infection with in one-month before surgery or routine follow-up were excluded. Follow-up was done as per NCCN guidelines. Fibrinogen and D-dimer levels were measured with in seven days of surgery or follow-up and analyzed for recurrence-free survival (RFS) and progression-free survival (PFS). Cox regression analyses were adopted to assess the influence of these two parameters on RFS and PFS.

**Results:**

The mean age was 53.9 years with a median follow-up of 9-months. Nine had recurrence of which six had progression. The cut-off values of fibrinogen and D-dimer were 402.5 mg/dl and 0.55 µg/ml, respectively. Kaplan–Meier analysis demonstrated that high fibrinogen and D-dimer levels were significantly related to poor RFS and PFS (*p* < 0.001). On multivariate analysis only fibrinogen and D-dimer retained their significance for RFS (*p* = 0.026 and 0.014, respectively) and PFS (*p* = 0.027 and 0.042, respectively). High levels of fibrinogen and D-dimer were also present in patients who had recurrence or progression at follow-up visits compared to rest of the patients.

**Conclusions:**

High levels of fibrinogen and D-dimer may indicate worse prognosis in patients with NMIBC, suggesting that these two can be used as prognostic biomarkers.

## Background

Bladder cancer is second most prevalent tumors of the genitourinary tract worldwide. Its incidence in developed countries (9.5 per 100,000) is almost three times higher than that in less developed countries (3.3 per 100,000) [1]. Approximately 70% of patients initially diagnosed with UCB have their tumor localized to mucosa and submucosa, known as non-muscle invasive bladder carcinoma (NMIBC) [2]. Such patients usually have a good prognosis, but there is risk of recurrence in 48–70% of the cases and progression to muscle invasiveness in about 27–61% of the cases [3, 4]. The standard therapeutical protocol that is followed for these patients includes transurethral resection of bladder tumor (TURBT), eventually combined with intravesical instillation of chemotherapeutic agents based upon risk stratification [5]. Owing to frequent relapse and high progression rates, active cancer surveillance using standard methods of diagnosis is important, which makes bladder cancer one of the most expensive cancer to manage. Several prognostic factors have been reported that predict the recurrence and progression in patients with NMIBC, such as serum cholinesterase, neutrophil to lymphocyte ratio, albumin to-globulin ratio, etc., to identify patients with high or low risk and tailor further management accordingly. However, till date, none of these factors have been able to accurately predict the risk of progression or recurrence, in patients with bladder cancer [6–8]. Various risk stratification systems have been developed such as EORTC risk tables, the Club Urológico Espanol de Tratamiento Oncológico (CUETO) scoring model, etc., to predict the probability of recurrence and progression but lacks independent external validation [9]. These scoring models use clinical and pathological variables to estimate the risk of recurrence and progression. But evaluation of some of these variables is subjective such as tumor size and number. All these inaccuracies may lead to an incorrect tumor classification, which makes the process of validation complicated [9–11]. Therefore, it is essential that some simple prognostic markers in NMIBC be identified so that better evaluation of tumor aggressiveness and its prognosis can be done, and accordingly accurate individualized treatment could be provided.

Several studies have confirmed that parameters of the coagulation/fibrinolysis system are abnormal in various cancers [12, 13]. Cao et al. reported that preoperative D-dimer levels can predict survival in patients with resectable pancreatic cancer [14]. Hou et al. observed that the number of elevated preoperative coagulation factors may have a significant effect on progression-free survival and could be used to predict the prognosis of non-small cell lung carcinoma patients after surgery [15]. The exact molecular mechanism of plasma fibrinogen and D-dimer by which they promote disease progression remains controversial. It has been observed that fibrinogen gets deposited around malignant tumor cells to form a protective shield against endogenous defense mechanisms. It also promotes angiogenesis, metastasis, and proliferation as well as potentiate the effect of growth factors such as the vascular endothelial growth factor and the fibroblast growth factor, on tumor cells [16]. It has also been reported that tumor cells themselves could promote conversion of fibrinogen into fibrin, thereby increasing their ability of vascular growth, proliferation, and invasion [17]. D-dimer is a specific cleavage product of fibrin and its high levels in plasma represents hyperfibrinolysis. High levels of plasma D-dimer have also been reported to indicate the existence of circulating tumor cells or micro-metastases and help in predicting high-risk pathological features of tumors [18]. 

However, the potential prognostic value of coagulation parameters, plasma fibrinogen and D-dimer, in predicting recurrence-free (RFS) and progression-free survival (PFS) in patients with NMIBC remains unknown. So, the purpose of our study was to evaluate prognostic significance of plasma fibrinogen and D-dimer in patients with NMIBC.

## Methods

All patients who presented for the first time with bladder mass between September 2020 and December 2021 were included in this prospective observational study after obtaining approval from the Institutional Ethics committee (803/Ethics/2020. Reference code: 101st ECM II B/P48). Informed written consent was taken from all the patients included in this study after explaining the nature of study. The selected patients met the following inclusion criteria: (1) presenting for first time with bladder mass; (2) the post-operative pathological results after TURBT consistent with NMIBC; (3) results of plasma fibrinogen and D-dimer levels available from within seven days before surgery and on follow-up; (4) a minimum follow-up duration of six months. The excluded patients had following characteristics: (1) incomplete resection (2) perioperative death (3) thromboembolic event within three months or congenital thrombophilia (4) anticoagulant or pro-coagulant therapy within the past eight-weeks (5) patients with deranged liver or renal functions (6) active infection, endocarditis, pregnancy, inflammatory bowel disease or serious refractory hypertension (7) patients who were diagnosed with COVID-19 infection within one-month before surgery or routine follow-up.

### Methodology

Clinical characteristics of patients, including age, sex, BMI, tobacco exposure and general physical examination were recorded. Patient’s fasting blood samples were collected within seven days before surgery and on follow-up to determine the levels of plasma fibrinogen and D-dimer. Biomarker levels were measured using an automatic analyzer (Stago STA Satellite®, France). Plasma fibrinogen and D-dimer were measured using the solidification and immune-turbidimetry method, respectively.

All patients underwent TURBT and intra-operative findings were recorded. The pathological diagnosis of tumor was established by a single pathologist, with > ten-years of experience in genitourinary malignancies, based on hematoxylin–eosin stained sections combined with immune-histochemical methods. Patient’s pathological characteristics including pathological stage and differentiation grade were recorded according to the UICC-TNM (2018) and WHO/ISUP (2004) classification, respectively.

Patients were followed up as per the NCCN-guidelines. The routine investigations at each follow-up consist of physical examination, blood investigations (including Plasma fibrinogen and D-dimer) and check cystoscopy. Excretory urogram and/or computed tomography (CT) was done to assess the upper urinary tract annually. The authors confirm the availability of, and access to all original data reported in this study.

### Statistical analysis

All prospective data was analyzed using IBM SPSS Statistics Windows, version 24.0 (Armonk, NY: IBM Corp.). The receiver operating characteristic (ROC) curve for preoperative D-dimer and plasma fibrinogen were built to evaluate the optimal cut-off values of the coagulation parameters. The relationship between the clinico-pathologic factors and the coagulation parameters was analyzed using Chi-square test and Fisher’s exact test. The RFS (the interval from primary TURBT until the first reoccurrence of disease) and the PFS (the interval from initial surgical therapy to first progress of disease i.e. any advance in tumor grade or stage) were evaluated by creating Kaplan–Meier curves. Variables showing significant impact on survival in univariate analysis (*p* ≤ 0.05) were included in the Cox proportional hazard regression models for multivariate analysis. A *p* < 0.05 was considered to be statistically significant.

## Results

A total of 66 patients were operated at our center from September 2020 to June 2021, who presented for the first time with a urinary bladder mass. Of these, 35 patients fulfilled the inclusion/exclusion criteria and were included in the study. Thirty were males and the mean age of subjects was 53.91-years (range, 27–81 years). The median follow-up was 9-months (range, 3–15-months). The demographic details are depicted in Table 1. Nine (25.71%) patients had disease recurrence, with progression to higher grade in two (5.7%) and invasion of muscle in four (11.4%).

**Table 1:** Demographic and baseline data of the study population

Parameter	Value
Total patients	35
Mean ± SD (range)	
Age (years)	53.91 ± 12.2 (27–81)
BMI (Kg/m^2^)	24.44 ± 2.76 (19.50–31.22)
Duration of symptoms (months)	5.83 ± 4.46 (0.25–18)
	N (%)
Gender	
Male	30 (85.7)
Female	05 (14.3)
Diet	
Vegetarian	20 (57.1)
Non-vegetarian	15 (42.9)
Presenting complaint	
Hematuria	34 (97.1)
Pain abdomen	01 (2.9)
Tobacco exposure	
Yes	24 (68.6)
No	11 (31.4)
Comorbidities	
None	29 (82.9)
Diabetes mellitus	02 (5.7)
Hypertension	02 (5.7)
Both	02 (5.7)
Size	
≤ 3 cm	24 (68.6)
> 3 cm	11 (31.4)
T-stage	
Ta or Tis	02 (5.7)
T1N0M0	33 (94.3)
Number	
Single	19 (54.3)
Multiple	16 (45.7)
Grade	
Low	26 (74.3)
High	09 (25.7)

### Determination of optimal cut-off values of plasma fibrinogen and D-dimer regarding prediction of RFS

**Fig. 1:**
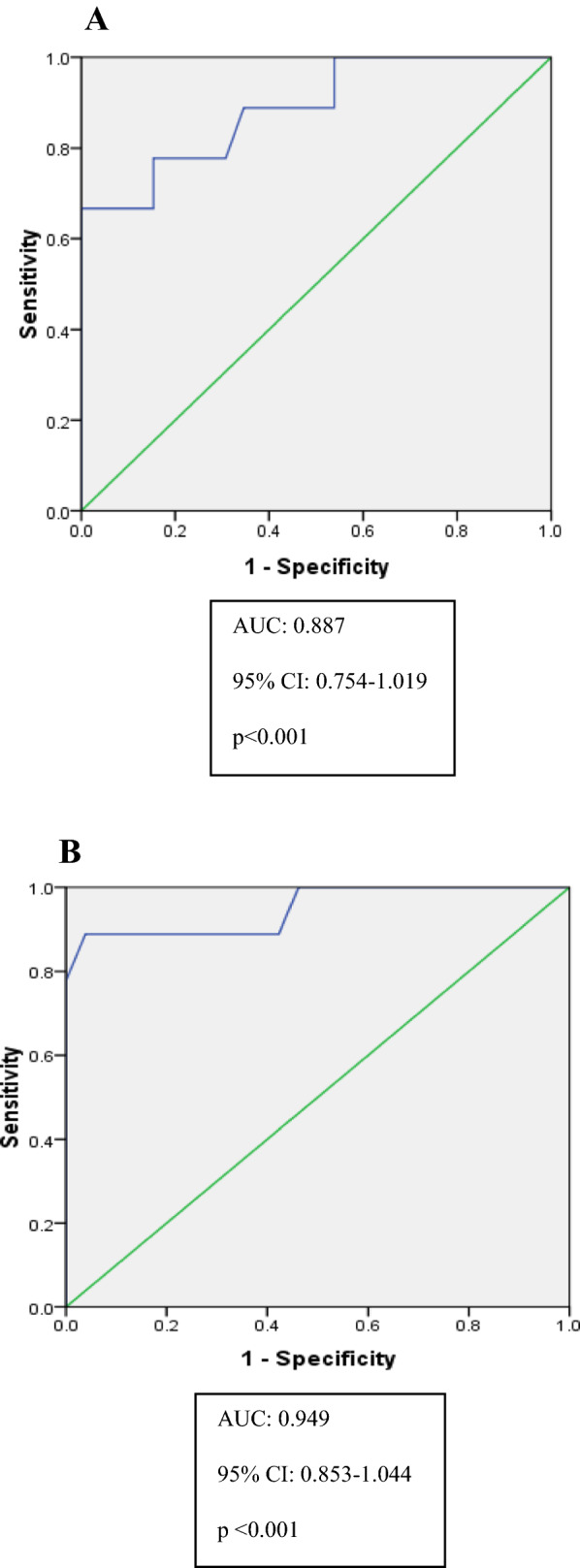
ROC curve analysis for determination of optimal cut-off values for pre-operative plasma fibrinogen (**A**) and D-dimer (**B**)

The mean value of pre-operative plasma fibrinogen and D-dimer was 351.19 mg/dl (range, 217–505 mg/dl) and 0.448 µg/ml (range, 0.13–1.40 µg/ml), respectively. Patients who had recurrence of disease, had higher mean value of pre-operative plasma fibrinogen and D-dimer (428.22 mg/dl and 0.72 µg/ml, respectively) compared to patients without recurrence (323.69 mg/dl and 0.36 µg/ml, respectively). ROC curves were drawn to obtain optimal cut-off values for pre-operative plasma fibrinogen and D-dimer, Fig. 1. The optimal cut-off value for plasma fibrinogen was 402.5 mg/dl (area under curve- 0.887, sensitivity 0.667, specificity 1.0) and D-dimer was 0.55 µg/ml (area under the curve 0.949, sensitivity 0.889, specificity 0.962). Patients were divided into two groups on the basis of these cut-off values for further analyses. High levels of plasma fibrinogen and D-dimer were present in six (17.1%) and nine (25.7%) patients, respectively, and low levels were present in 29 (82.9%) and 26 (74.3%) patients, respectively.

### Relationship between clinico-pathologic characteristics and plasma fibrinogen and D-dimer in patients with NMIBC

Pre-operative plasma fibrinogen and D-dimer levels of patients were compared with demographic and clinico-pathological characteristics. Statistically, significantly higher levels of plasma fibrinogen and D-dimer were found in patients with larger tumor size (> 3 cm) compared to those with tumor size ≤ 3 cm (*p* < 0.001). No significant association was recorded with any other characteristic of patients, including BMI (*p* = 0.983) or the duration of symptoms (*p* = 0.839) (Table 2).

**Table 2:** Relationship between patient’s demographic, clinico-pathological characteristics and coagulation parameters

		Plasma fibrinogen (Mg/Dl)				*p*-value	D-dimer (µg/ml)				*p*-value
		≤ 402.50		> 402.50			≤ 0.55		> 0.55		
		N	%	N	%		N	%	N	%	
Age*	≤ 55 years	16	55.2	2	33.3	0.402	14	53.8	4	44.4	0.711
	> 55 years	13	44.8	4	66.7		12	46.2	5	55.6	
Gender	Male	25	86.2	5	83.3	1.000	22	84.6	8	88.9	1.000
	Female	04	13.8	1	16.7		04	15.4	1	11.1	
Tobacco exposure	No	10	34.5	1	16.7	0.640	08	30.8	3	33.3	0.886
	Yes	19	65.5	5	83.3		18	69.2	6	66.7	
Grade	Low	21	72.4	5	83.3	1.000	19	73.1	7	77.8	0.781
	High	08	27.6	1	16.7		07	26.9	2	22.2	
Size	≤ 3 cm	23	79.3	1	16.7	0.007	23	88.5	1	11.1	< 0.001
	> 3 cm	06	20.7	5	83.3		03	11.5	8	88.9	
T stage	Ta or Tis	02	6.9	0	0.0	1.000	02	7.7	0	0.0	1.000
	T1NoMo	27	93.1	6	100.0		24	92.3	9	100.0	
Number	Single	16	55.2	3	50.0	1.000	15	57.7	4	44.4	0.700
	Multiple	13	44.8	3	50.0		11	42.3	5	55.6	

*Median age-55 years

### Relationship between plasma fibrinogen, D-dimer and RFS in patients of NMIBC

**Fig. 2:**
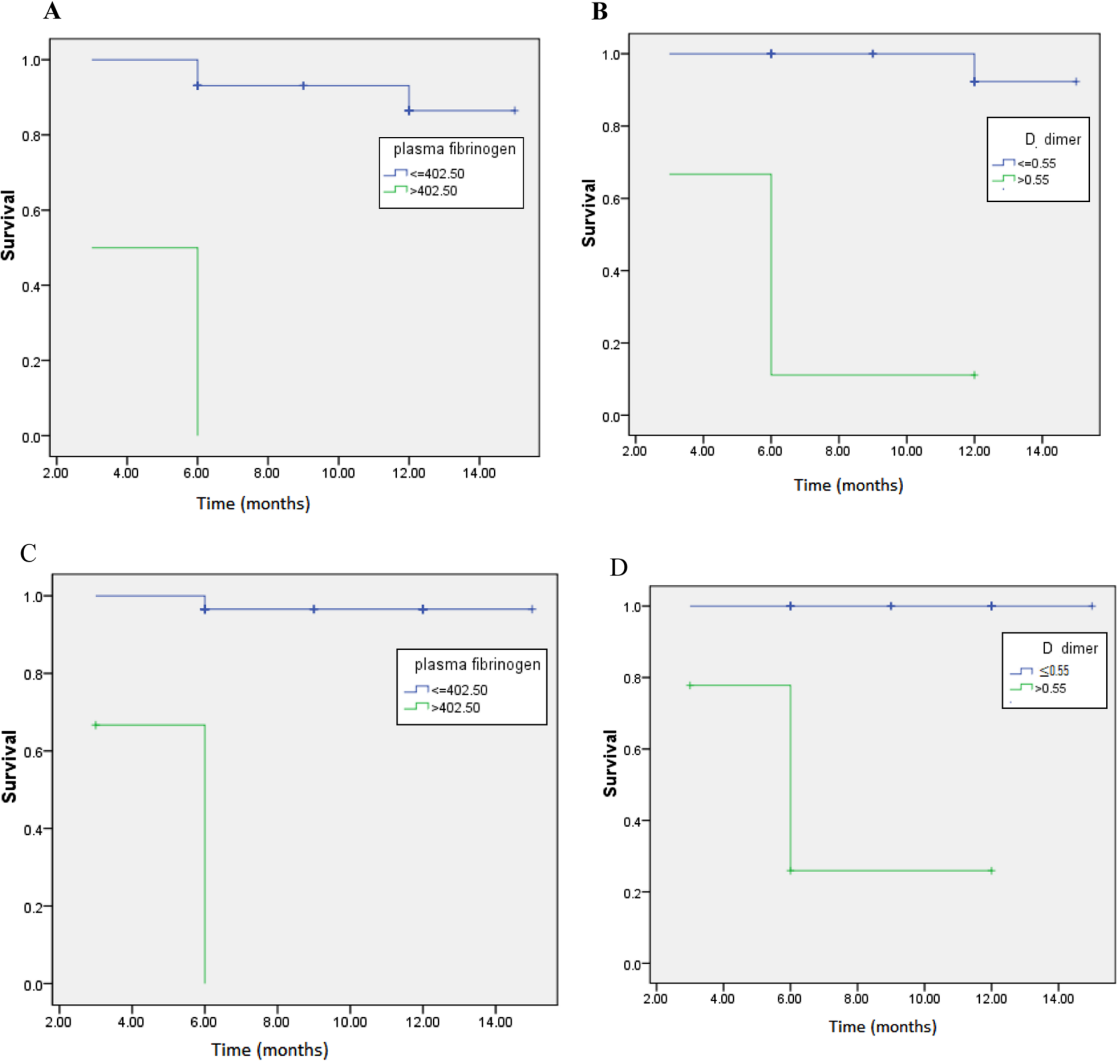
Kaplan–Meier curves for RFS (**A**, **B**) and PFS (**C**, **D**) for NMIBC patients stratified by preoperative plasma fibrinogen and D-dimer

To assess the potential prognostic value of plasma fibrinogen and D-dimer in predicting RFS, Kaplan–Meier survival analysis was carried out. The results demonstrated that patients with plasma fibrinogen level > 402.5 mg/dl (*p* < 0.001) and D-dimer level > 0.55 mcg/ mL (*p* < 0.001) had significantly poorer RFS compared to those with lower levels of both the parameters, Fig. 2. Univariate and multivariate analysis was done using Cox proportional hazards model to find any significance between patient’s demographic characteristics, clinico-pathological parameters, pre-operative plasma fibrinogen and D-dimer, and risk of recurrence or progression of disease. Three parameters, including tumor size, pre-operative plasma fibrinogen and D-dimer, were found to be related to RFS on univariate analysis but on multivariate analysis only two parameters, high levels of pre-operative plasma fibrinogen and D-dimer were found to have independent association with a shorter RFS (*p* = 0.026 and 0.014, respectively) (Table 3).

**Table 3:** Univariate and multivariate analyses of prognostic factors for disease recurrence by cox proportional hazards model

Characteristic		Univariate analysis		Multivariate analysis	
		HR (95% CI)	*p*-value	HR (95% CI)	*p*-value
Age*	≤ 55 years	2.341 (0.583–9.399)	0.230	–	–
	> 55 years				
Gender	Male	0.802 (0.099–6.518)	0.836	–	–
	Female				
Tobacco exposure	No	1.767 (0.367–8.513)	0.478	–	–
	Yes				
Grade	Low	0.331 (0.041–2.651)	0.298	–	–
	High				
Post-op intravesical therapy	No	0.517 (0.158–2.530)	0.517	–	–
	Yes				
Size	≤ 3 cm	8.888 (1.840–42.930)	0.007	0.294 (0.029–3.034)	0.304
	> 3 cm				
T stage	Ta or Tis	22.133 (0.000–3.672)	0.614	–	–
	T1N0M0				
Number	Single	1.419 (0.381–5.287)	0.602	–	–
	Multiple				
Urine Cytology (pre-operative)	Positive	1.901 (0.870–1.380)	0.437	–	–
	Negative				
Pre-operative Plasma Fibrinogen (mg/dl)	≤ 402.50	23.007 (4.574–115.730)	< 0.001	3.126 (0.514–18.997)	0.026
	> 402.50				
Pre-operative D-dimer (µg/ml)	≤ 0.55	37.063 (4.489–305.983)	0.001	61.618 (2.304–16.48)	0.014
	> 0.55				

*Median age-55 years

### Plasma fibrinogen, D-dimer and progression-free survival (PFS) of NMIBC patients

To assess the potential prognostic value of plasma fibrinogen and D-dimer in predicting PFS, Kaplan–Meier survival analysis was carried out. The results demonstrated that patients with plasma fibrinogen level more than 402.5 mg/dl (*p* < 0.001) and D-dimer level more than 0.55 mcg/ mL (*p* < 0.001) had significantly poorer PFS compared to those with lower levels of both the parameters (Fig. 1). Univariate and multivariate analysis was done using Cox proportional hazards model to find any significance between patient’s demographic characteristics, clinico-pathological parameters, coagulation parameters (pre-operative plasma fibrinogen and D-dimer) and risk of recurrence or progression of disease. Three parameters, including, tumor size, pre-operative plasma fibrinogen and D-dimer, were found to be related to PFS on univariate analysis but on multivariate analysis only two parameters, high levels of pre-operative plasma fibrinogen and D-dimer, were found to have independent association with a shorter PFS (*p* = 0.027 and 0.042 respectively) (Table 4).

**Table 4:** Univariate and multivariate analyses of prognostic factors for disease progression by cox proportional hazards model

Characteristic		Univariate analysis		Multivariate analysis	
		HR (95% CI)	*p*-value	HR (95% CI)	*p*-value
Age*	≤ 55 years	1.108 (0.224–5.490)	0.900	–	–
	> 55 years				
Gender	Male	1.118 (0.131–9.570)	0.919	–	–
	Female				
Tobacco exposure	No	2.508 (0.293–21.482)	0.401	–	–
	Yes				
Grade	Low	0.532 (0.062–4.554)	0.564	–	–
	High				
Post-op intravesical therapy	No	1.301 (0.263–6.448)	0.747	–	–
	Yes				
Size	≤ 3 cm	12.144 (1.418–104.030)	0.043	1.248 (0.056–27.693)	0.889
	> 3 cm				
T stage	Ta or Tis	22.090 (0.000–6.119)	0.683	–	–
	T1N0M0				
Number	Single	1.151 (0.232–5.704)	0.863	–	–
	Multiple				
Urine Cytology (pre-operative)	Positive	3.095 (0.361–26.497)	0.302	–	–
	Negative				
Pre-operative Plasma Fibrinogen (mg/dl)	≤ 402.50	30.232 (4.141–220.720)	0.001	33.726 (1.507–754.825)	0.027
	> 402.50				
Pre-operative D-dimer (µg/ml)	≤ 0.55	917.840 (0.004–2.240)	0.028	5.590 (0.000–5.912)	0.042
	> 0.55				

*Median age 55 years

### Relationship between RFS, PFS and coagulation parameter’s level at follow up

It was also observed that mean plasma fibrinogen and D-dimer values were significantly higher at 3-month and 6-month follow-up in patients who had recurrence (*p* < 0.001) or progression (*p* < 0.001) of disease compared to those who had neither recurrence nor progression (Table 5)**.**

**Table 5:** Comparison between mean plasma D-dimer levels with recurrence and progression at 3 and 6 months follow-up

	No recurrence	Recurrence	Progression
Mean D-Dimer at 3 months (µg/ml)	0.39	0.64	0.69
Mean D-Dimer at 6 months (µg/ml)	0.38	0.58	0.66

## Discussion

Urothelial carcinoma accounts for more than 90% of all the bladder tumors [19]. Majority of new patients, who present with bladder mass are initially diagnosed with NMIBC, which frequently recurs [3, 4]. Also, the patients who progress to muscle invasive disease have a poorer prognosis compared to those who had MIBC at initial presentation [20]. Thus, it is important to identify factors that can predict high risk disease in NMIBC patients, so that appropriate risk adapted treatment and surveillance strategies could be employed in such patients for early detection and timely management.

A total of 66 new patients with bladder mass were operated in our institute from September 2020 to June 2021, but only 35 patients were eligible for inclusion into the study. Similar to other large studies there was a clear male preponderance (30 versus 5) [21, 22]. 

Age is a strong and independent risk factor for the development of UCB [23]. The mean age at presentation was 53.91-years (SD ± 12.2; range, 27–81). No significant difference was noticed in the histological grade of tumor among patients of different age groups.

Various demographic and clinico-pathological characteristics including smoking, increased BMI, high tumor grade, large size (> 3 cm), multiple number and T-stage have been associated with increased risk of bladder tumor recurrence and progression [4, 24, 25]. In our study, only tumor size (> 3 cm) was found to be significantly associated with increased risk of recurrence and progression on univariate analysis (*p* = 0.007 and 0.043, respectively); however, on multivariate analysis, it failed to sustain independent association. No statistically significant relationship was found among rest of the demographic or clinico-pathological characteristics with the risk of recurrence or progression. The reason could be small sample size.

Numerous studies have demonstrated that the coagulation/fibrinolytic system is initiated in vivo in patients with various cancers and the levels of these parameters can be used to predict tumor load and prognosis [14, 15]. In another study, Chen et al. reported that elevated plasma D-dimer levels (> 0.36 mg/L) were significantly associated with poorer survival in patients with upper tract urothelial carcinoma. They also reported that pre-operative D-dimer levels can help in improving the risk stratification and a more accurate prediction of recurrence and survival. This can help in decision making for follow-up scheduling, administration of adjuvant therapies, and precise individualized treatment for patients [26]. In another study on prognostic value of preoperative inflammation-based predictors in patients with bladder carcinoma by Gui et al., they reported that high plasma fibrinogen levels are associated with a more aggressive clinical stage and plasma fibrinogen levels may be used to predict survival outcome of patients with bladder carcinoma [27]. 

In our study, we found that high levels of pre-operative plasma fibrinogen (> 402.5 mg/dl) and D-dimer (> 0.55 µg/ml) were present in 13 (37.1%) and 10 (28.6%) patients and low levels were present in 22 (62.9%) and 25 (71.6%) patients, respectively. When compared with patient’s demographic and clinic-pathological characteristics, statistically significant higher levels of pre-operative plasma fibrinogen (> 402.5 mg/dl) and D-dimer (> 0.55 µg/ml) were found in patients with larger tumor size (> 3 cm) compared to those with smaller tumor size (*p* < 0.001). Higher levels of pre-operative plasma fibrinogen (> 402.5 mg/dl) and D-dimer (> 0.55 µg/ml) were not found to be significantly associated with any other demographic or clinico-pathological characteristic of patient.

On multivariate analysis, it was also observed that patients with recurrent disease and those who had progression, had significantly higher levels of the pre-operative plasma fibrinogen (> 402.5 mg/dl; *p* = 0.026 and 0.027, respectively) and D-dimer (> 0.55 µg/ml; *p* = 0.014 and 0.042, respectively) compared to those who had no recurrence or progression of disease within the follow-up period. To assess the potential prognostic value of these two coagulation parameters (plasma fibrinogen and D-dimer) in predicting RFS and PFS, Kaplan–Meier survival analysis was performed. The results demonstrated that patients with higher levels of plasma fibrinogen (> 402.5 mg/dl) and D-dimer (> 0.55 mcg/ mL) had significantly shorter RFS (*p* < 0.001) and PFS (*p* < 0.001) compared to those with lower levels of both the parameters. These results were similar to the study by Li et al. that demonstrated that NMIBC patients who had high plasma fibrinogen (> 3.56 g/L) and D-dimer (> 0.48 mg/mL) levels, had worse pathologic features (T-stage, tumor size and multiplicity of tumor) as well as significantly poor RFS (*p* = 0.029 and 0.001, respectively) and PFS (*p* = 0.023 and 0.003, respectively) [28]. 

In our study, we also found that mean plasma fibrinogen and D-dimer value was significantly higher at 3-month and 6-month follow-up in patients who had recurrence (*p* < 0.001) or progression (*p* < 0.001) of disease compared to those who had neither recurrence nor progression. But a statistically significant association could not be established for mean plasma fibrinogen values at 9-months and 12-month follow-up because of very small number of patients. After thorough literature search, we could not find any study comparing the levels of plasma fibrinogen and D-dimer at follow-up visits with the risk of recurrence and progression. In our study too, definitive inference could not be drawn due to a very small sample size.

Our study has several limitations. First, the surgeons performing the surgery were heterogeneous with residents in training also performing many TURBT under supervision of trained consultant urologists. Second, the duration of follow-up is very short with a maximal follow-up of 15-months. Lastly, the sample size was relatively small. Therefore, larger prospective studies are needed to further consolidate the position of plasma fibrinogen and D-dimer in predicting disease recurrence and progression in patients with NMIBC and help in making individualized risk-adapted treatment decisions.

## Conclusions

High levels of plasma fibrinogen and D-dimer may serve as prognostic factors in patients with NMIBC to predict RFS and PFS. Patients with higher risk of recurrence or progression have higher levels of plasma fibrinogen and D-dimer at subsequent follow-up also. These coagulation parameters can help in making individualized risk-adapted treatment decisions and surveillance strategies. A further study with longer follow-up and larger sample size is required for better interpretation of result.

## Abbreviations

AR: Androgen receptor
ATF: Activating transcription factors
BC: Bladder cancer
BMI: Body mass index
EMT: Epithelial mesenchymal transition
MIBC: Muscle invasive bladder carcinoma
MMC: Mitomycin-C
NLR: Neutrophils to lymphocyte ratio
NMIBC: Non-muscle invasive bladder carcinoma
PFS: Progression-free survival
RFS: Recurrence-free survival
SCC: Squamous cell carcinoma
SUO: Society of urologic oncology
TURBT: Transurethral resection of bladder tumor
UCB: Urothelial carcinoma of bladder
UTUC: Upper tract urothelial carcinoma
WLC: White light cystoscopy
